# Involvement of the G-Protein-Coupled Receptor 4 in the Increased Expression of RANK/RANKL/OPG System and Neurotrophins by Nucleus Pulposus Cells under the Degenerated Intervertebral Disc-Like Acidic Microenvironment

**DOI:** 10.1155/2020/1328436

**Published:** 2020-05-29

**Authors:** Hao Li, Huafei Liu, Ning Zhang, Zemin Zhu

**Affiliations:** ^1^Department of Orthopedics, 2nd Affiliated Hospital, School of Medicine, Zhejiang University, Hangzhou, China; ^2^Department of Orthopaedics, Changxing District of 2nd Affiliated Hospital, School of Medicine, Zhejiang University, Huzhou, China

## Abstract

Intervertebral disc (IVD) degeneration is associated with local inflammation and increased expression of neurotrophins. Acidic microenvironment is believed to cause the progression of IVD degeneration. However, there is a paucity of information regarding the relationship between acidic microenvironment and the inflammation and expression of neurotrophins in IVD. G-protein-coupled receptor 4 (GPR4) is a pH-sensing receptor, which can activate the inflammation and increase the expression levels of nerve growth factor in acidic microenvironment. In this study, culture media with pH 7.2 (representing the normal IVD-like acidic condition) and pH 6.5 (degenerated IVD-like acidic condition) were prepared. The gene and protein expression levels of GPR4 in SD rat nucleus pulposus cells were determined under the acidic conditions. And cyclic AMP (cAMP), the second messenger of GPR4, was assayed. Furthermore, the expression levels of receptor activator of nuclear factor *κ* B (RANK), RANKL ligand (RANKL), osteoprotegerin (OPG), nerve growth factor (NGF), brain-derived neurotrophic factor (BDNF), and neurotrophin-3 (NT-3) were also determined. To clarify the involvement of GPR4 in the upregulation of the expression of RANK/RANKL/OPG system and neurotrophins, gene knockdown and forced expression of GPR4 and inhibiting its downstream cAMP accumulation and Ca^2+^ mobilization were performed. The alternation of the expression levels of matrix metalloproteinase-3 (MMP-3), MMP-13, and aggrecanase-2 (ADAMTS-5) were evaluated by RT-PCR and western blot. The results showed that GPR4 was expressed in rat nucleus pulposus cells, and the expression was upregulated under the degenerated IVD-like acidic microenvironment. cAMP accumulation levels were increased under the degenerated IVD-like acidic culture conditions. The expression levels of RANK, RANKL, OPG, NGF, and BNDF were significantly upregulated under the degenerated IVD-like acidic microenvironment. GPR4 knockdown and reduction of cAMP by the inhibitor SQ22536 abolished the upregulation of the expression of RANK, RANKL, OPG, NGF, and BNDF under the degenerated IVD-like acidic microenvironment. On the opposite, acidosis-induced cAMP accumulation and upregulation of RANK, RANKL, OPG, NGF, and BNDF were further promoted by GPR4 overexpression. The expression levels of MMP-3, MMP-13, and ADAMTS-5 were upregulated under the degenerated IVD-like acidic condition, which can be promoted or attenuated by GPR4 overexpression or knockdown, respectively. We concluded that GPR4-mediated cAMP accumulation was involved in the increased expression of RANK/RANKL/OPG system and neurotrophins by nucleus pulposus cells under the degenerated IVD-like acidic microenvironment.

## 1. Introduction

Low back pain is one of the most common and costly musculoskeletal problems worldwide, which increases a severe economic burden to the patients and the society [1, 2]. Intervertebral disc (IVD) degeneration is accepted as the most important reason for low back pain. Many in vitro and in vivo studies evidenced that several pathological changes appear in the degenerated discs, including local inflammation, imbalance in the extracellular matrix metabolism, and sensitizing innervation into the disc [3–5].

Although the specific molecular mechanism of IVD degeneration remains elusive, local inflammation [3, 6] and elevated expression of neurotrophins [5, 7] have been identified as important players in the progression of disc degeneration. Tremendous amount of studies evidenced the abnormal expression of proinflammatory cytokines in the disc contribute to upregulate the expression of matrix-degrading enzymes [8, 9]. For example, receptor activator of NF-*κ*B ligand (RANKL) is a member of the tumor necrosis factor ligand superfamily, and it is known that RANKL, its signaling receptor (RANK), and its decoy receptor osteoprotegerin (OPG) are key regulators of the production of extracellular matrix remodeling enzymes [10]. Recently, the expression level of the RANK/RANKL/OPG system was found to be upregulated in degenerated human IVD tissues and be associated with in the process of IVD degeneration [11]. More importantly, the upregulation of RANK/RANKL/OPG can result in the biosynthesis of extracellular matrix-degrading enzymes, including matrix metalloprotease-3 (MMP-3) and MMP-13 in degenerated IVD [11]. On the other hand, neurotrophin is another biological contributor for the progression of IVD degeneration [12]. Among the several neurotrophins, nerve growth factor (NGF), brain-derived neurotrophic factor (BDNF), and neurotrophin-3 (NT-3) are the most studied factors, which are essential in neuronal development and modulation of pain [13, 14]. It is reported that neurotrophins could be generated by IVD cells as well as immune cells, which lead to the innervation of aneural regions of the disc [7]. The increased expression of neurotrophins in severely degenerate discs has been implicated in chronic low back pain associated with the progression of disc degeneration [15, 16].

The microenvironment of degenerated IVD is unique and hostile, mainly characterized with acidosis when compared with the pH level in the normal internal environment of the human body (pH 7.3 to 7.5). For example, the pH range is 7.0 to 7.2 in healthy disc and 6.5 to 6.8 in the mild degenerative IVD, which is lower than the pH in the normal internal environment of the human body [17]. In a previous study, acid-sensing ion channels (ASICs), a sort of voltage-insensitive Na^+^ channels, were reported to play critical roles in physiological and pathological conditions of the disc [18, 19]. And acidic microenvironment has been reported to be associated with the local inflammation [20] and upregulation of neurotrophins [21], which leads to the progression of IVD degeneration. However, the underlying molecular mechanism is not fully understood.

G-protein-coupled receptor (GPR) 4 is a proton-sensing G-protein-coupled receptor that senses extracellular acidic pH and stimulate several intracellular signaling pathways, participating in inflammation, angiogenesis, and oncogenesis [22–24]. In previous studies, GPR4 was found to be involved in increasing the expression of RANK/RANKL/OPG under acidic conditions in osteoblastic cells [25] and increasing the sensitivity of nociceptors of dorsal root ganglion [26]. However, there was no study about the regulation role of GPR4 on the expression of RANK/RANKL/OPG and neurotrophins in nucleus pulposus cells under the degenerated IVD-like acidic microenvironment. Based on these previous studies, we hypothesized that GPR4 may be involved in the regulation of the expression of RANK/RANKL/OPG and neurotrophins by nucleus pulposus cells under the degenerated IVD-like acidic microenvironment. Therefore, the aim of the current study was to investigate (1) the effect of the degenerate IVD-like acidic microenvironment on the expression of RANK/RANKL/OPG and the neurotrophin production of nucleus pulposus cells under the degenerate IVD-like acidic microenvironment; (2) determine the involvement of GPR4 and its second messenger molecular cAMP in these processes.

## 2. Materials and Methods

### 2.1. Nucleus Pulposus Cell Isolation and Culture

The research was approved by the Ethics Committee of the second affiliated hospital of Zhejiang University (No: 2015-038). All of the experiments were performed in accordance with the ethical standards. A total of 8 SD rats (2 months old) were provided by the Experimental Animal Center of Zhejiang University (Hangzhou, China). Lumbar IVDs were harvested from the rats immediately following sacrifice (intraperitoneal injection of excessive 0.3% sodium pentobarbital). Under a stereotaxic microscope, nucleus pulposus was carefully separated from the annulus fibrosus and cut into small fragments (2 mm^3^). Nucleus pulposus cells were isolated by enzymatic digestion for 12 h at 37°C in Dulbecco's modified Eagle's medium (DMEM)/F12 (Gibco, Grand Island, NY, USA) with 2% type II collagenase (Invitrogen, Carlsbad, CA, USA). Undigested tissues were removed and the nucleus pulposus cells were resuspended in DMEM/F12 containing 10% fetal bovine serum (Gibco), 2 mM Glutamax (Gibco), and 1% penicillin-streptomycin and incubated at 37°C in a humidified 5% CO_2_ atmosphere. When nucleus pulposus cells grew to 80% confluence, they were detached by trypsinization and cultured in 25 cm^2^ culture flasks. The culture medium was completely replaced every 3 days, and cells from passages 3 to 6 were harvested for the subsequent experiments. In order to analyze the downstream molecules involved in the regulation of GPR4, nucleus pulposus cells were pretreated for 72 h with SQ22536 (500 *μ*M) or U73122 (20 *μ*M).

### 2.2. Degenerated IVD-Like Acidic Microenvironment Mimicking Experiments

Degenerated IVD-like acidic microenvironment mimicking experiments were carried out in DMEM/F12, supplemented with 10% fetal bovine serum, 2 mM Glutamax, 1% penicillin-streptomycin, and 20 mM HEPES. The pH was adjusted using a calibrated pH meter (Metrohm, Herisau, Switzerland) with sterilized HCl (1 M), and the medium was equilibrated in a 5% CO_2_ incubator for 24 h. The culture media were prepared at pH 7.2 (representing the acidic microenvironment of normal IVD) and pH 6.5 (acidic microenvironment of degenerated IVD). All data presented are referenced to pH measured at room temperature.

### 2.3. Real-Time Quantitative Polymerase Chain Reaction (RT-PCR)

RT-PCR was performed after 24 h incubation in the acidic conditions. RNA was extracted using TRIzol reagent (TAKARA, Dalian, China) as previously described [27]. Total RNA was reversely transcripted to cDNA utilizing a Double-Strand cDNA Synthesis Kit (TAKARA) according to the manufacturer's instructions. SYBR Green PCR assays (TAKARA) were used to perform real-time PCR in StepOnePlus (Applied Biosystems, USA). Three independent samples were set to ensure validity. 18S rRNA was used as internal control, and target genes were detected (Table 1). Primers were synthesized by Sangon Biotech (Shanghai, China), and quantitative real-time PCR data were calculated by the 2^−*ΔΔ*Ct^ method. PCR assays were conducted at least three times in triplicates for each sample.

### 2.4. Gene Knockdown and Overexpression of GPR4

For GPR4 knockdown, three pairs of short hairpin RNA-coding sequences directed against different sites of GPR4 mRNA (Table 2) and a scrambled fragment (Cyagen, Guangzhou, China) were designed and inserted into lentiviral vector plasmid pLV [shRNA]-EGFP:T2A:Puro-U6 (Cyagen). These plasmids and lentiviral packaging plasmids were cotransfected into HEK293FT cells (Cell bank of the Chinese Academy of Sciences, Shanghai, China) using the Lipofectamine 2000 reagent (Invitrogen). Forty-eight hours after transduction, a virus containing conditioned media was harvested. Nucleus pulposus cells were infected with the filtered viral supernatant and polybrene (10 mg/mL; Sigma). GPR4 knockdown nucleus pulposus cells and empty vector (EV) introduced cells were established by puromycin selection for 1 week. Nucleus pulposus cells with the highest knockdown efficiency were named GPR4-KD cells.

For GPR4 overexpression, DNA fragments encoding full-length rat GPR4 (GenBank ID: NM_001025680) were amplified by a PCR-based technique using proofreading DNA polymerase (ExTaq polymerase, TAKARA, Japan). The primers used for cloning were 5′-TAGCCTGCCACAAAGCAAAC-3′ (forward) and 5′-CTGTCCTTATCTGCCAGAAACC-3′ (reverse). The GPR4 cDNA was then cloned into: pLV[Exp]-EGFP:T2A:Puro-EF1A. The constructs of pLV[Exp]-EGFP:T2A:Puro-EF1A-rGpr4 were verified by analysis of restriction enzyme digests and DNA sequencing. The GPR4 expression vector and pCL10A1 (a retroviral packaging vector, IMGENEX, San Diego, CA, USA) were cotransfected into nucleus pulposus cells using the Lipofectamine 2000 reagent. Forty-eight hours after transduction, a virus containing conditioned media was harvested. Nucleus pulposus cells were infected with the viral supernatant with polybrene (10 mg/mL). Nucleus pulposus cells overexpressing GPR4 were established by puromycin selection for 1 week, and were named GPR4-OE cells.

### 2.5. Western Blot Test

Western blot analysis was performed after 24 h incubation in the acidic conditions. Briefly, cells were washed three times with ice-cold PBS, and total proteins were extracted with RIPA buffer containing 1% PMSF. Protein concentrations were measured using a BCA Protein Quantification Kit (Takara). Proteins were electrophoresed by 8-12% sodium dodecyl sulfate polyacrylamide gel electrophoresis (SDS-PAGE) and then transferred onto polyvinylidene fluoride (PVDF) membranes (Millipore, Massachusetts, MA, USA). After blocking with 5% skim milk in Tris-buffered saline with 0.1% Tween-20 (TBST) at room temperature for 1 h, membranes were maintained overnight at 4°C in TBST with the primary antibodies. The membranes were then incubated with horseradish peroxidase- (HRP-) labeled secondary IgG (1 : 1000, Santa Cruz) for 1 h at room temperature. After the membrane washed three times with TBST, the immunoreactivity was detected with enhanced chemiluminescence (ECL, Millipore) substrate, and the densitometry was performed using Quantity One Software (Bio-Rad Laboratories Inc., Munich, Germany). GAPDH served as an internal control. Primary antibodies used in this experiment were anti-GPR4 (Abcam ab 75330), anti-RANK (NB100-565080), anti-OPG(Abcam ab73400), anti-NGF(Abcam ab52918), anti-BDNF(Abcam ab205067), anti-COX-2(Abcam ab52237), anti-MMP-3(Abcam ab52915), anti-MMP-13(Abcam ab84594), anti-aggrecanase-2(ADAMTS-5) (Abcam ab41037), and anti-GAPDH antibody (Abcam ab181602). Each experiment was repeated three times.

### 2.6. Intracellular cAMP Assay

After 24 h culture in the acidic conditions, cAMP accumulation levels in nucleus pulposus cells were evaluated using a colorimetric cAMP ELISA kit (Enzo Life Sciences, Farmingdale, NY, USA), in accordance with the manufacturer's instructions. In brief, nucleus pulposus cells were homogenized in 0.1 N HCl for 10 min, then centrifuged at 1000× g for 5 min. Finally, 100 *μ*L of supernatant was then transferred for the ELISA assay. The intracellular cAMP levels were determined via estimation of the optical density at 410 nm with a Tecan plate reader (Tecan Spectrafluor Plus). Results were standardized to the protein content present in the cell lysate as performed by BCA assay.

### 2.7. Statistical Analysis

Data are expressed as mean ± standard deviation (SD) or mean ± standard error of the mean (SEM) of at least three independent experiments. A one-way analysis of variance (ANOVA) with Tukey's post hoc adjustment and Student's *t*-test were used to assess differences. Statistical analyses were performed with SPSS 17.0 for Windows. Statistical significance was set at *P* < 0.05.

## 3. Results

### 3.1. GPR4 Expression and cAMP Accumulation Upregulated in Degenerated IVD-Like Acidic Condition

**Figure 1:**
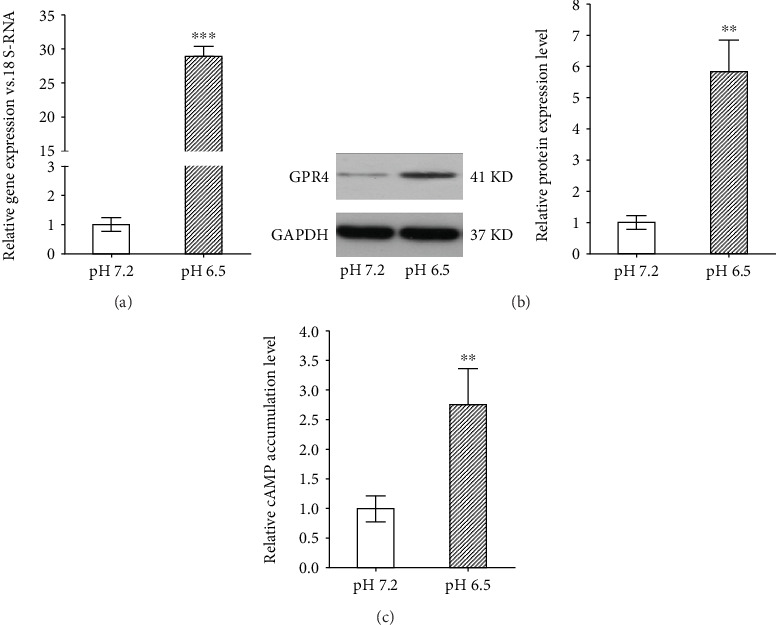
The expression of GPR4 and cAMP accumulation in the degenerated IVD-like acidic condition. The expression level of GPR4 in nucleus pulposus cells was determined by RT-PCR and western blot test (a, b) in pH 7.2 and pH 6.5. cAMP accumulation in nucleus pulposus cells was measured in pH 7.2 and pH 6.5 (C). Data were represented as mean ± SE of 6 independent experiments performed in triplicate; Student's *t*-test were used to find out differences. (^∗∗^*P* < 0.01 VS. control in pH 7.2; ^∗∗∗^*P* < 0.001 VS. control in pH 7.2).

**Figure 2:**
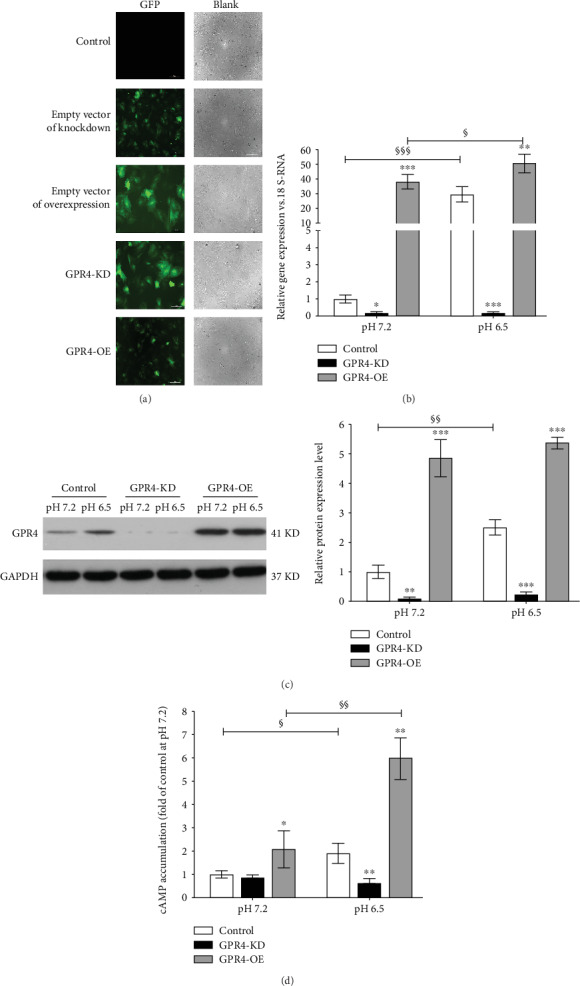
The influence of GPR4 knockdown and overexpression in the expression of GPR4 and cAMP accumulation in the degenerated IVD-like acidic condition. Lentiviral vectors, including empty vehicle, shRNA, and DNA fragments encoding full-length rat GPR4, were applied for the knockdown and overexpression of GPR4, and successful transduction was verified on the GFP expression (a), the mRNA level (b), and protein level (c). cAMP accumulation in nucleus pulposus cells with GPR4 knockdown and overexpression was measured in pH 7.2 and pH 6.5 (d). Data were represented as mean ± SE of 6 independent experiments performed in triplicate; ANOVA was used to find out differences between cells in the same pH level and Student's *t*-test was used to find out differences within the same cell between pH 7.2 and 6.5. (GPR4 KD: GPR4 knockdown; GPR4 OE: GPR4 overexpression; ^∗^*P* < 0.05; ^∗∗^*P* < 0.01; ^∗∗∗^*P* < 0.001; ^§^*P* < 0.05; ^§§^*P* < 0.01; ^§§§^*P* < 0.001).

As shown in Figure 1, the result of PCR and western blot demonstrated that GPR4 was expressed in nucleus pulposus cells. In addition, the gene and protein expression levels of GPR4 were relatively low in normal IVD-like acidic condition, which were increased 30.3 ± 8.8 fold (*P* < 0.001) and 5.9 ± 0.9 fold (*P* < 0.01) in degenerated IVD-like acidic condition. Meanwhile, the intracellular cAMP level was elevated 2.8 ± 0.4 fold (*P* < 0.01) in the degenerated IVD-like acidic condition. To further evaluate the potential role of GPR4, GPR4 gene was knocked down or forced expression. Successful transduction was confirmed by fluorescent signals of GFP, and gene amplification and protein expression (Figure 2(a)–2(c)). We found that GPR4 knockdown inhibited the cAMP accumulation (34.2 ± 10.1%, *P* < 0.05) in the degenerated IVD-like acidic condition when compared to the control group, on the opposite GPR4 overexpression further promoted the elevation of the cAMP accumulation (3.2 ± 0.7 fold, *P* < 0.05) (Figure 2(d)).

### 3.2. Involvement of GPR4 in the Upregulation of RANK/RANKL/OPG in Degenerated IVD-Like Acidic Condition

**Figure 3:**
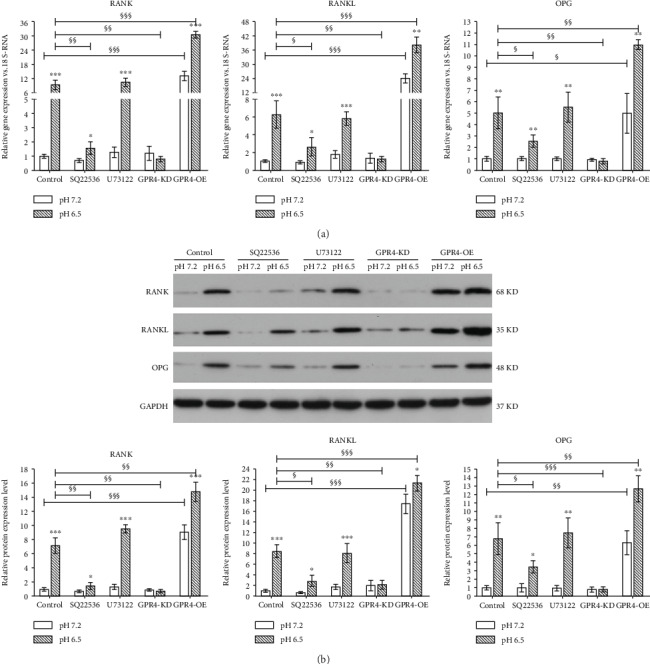
The influence of GPR4 and its downstream signaling molecules in the expression levels of the receptor activator of NF-*κ*B (RANK), RANK ligand (RANKL), and osteoprotegerin (OPG) in the degenerated IVD-like acidic condition. The expression levels of RANK, RANKL, and OPG were assayed by RT-PCR (a) and western blot (b). Data were represented as mean ± SE of 6 independent experiments performed in triplicate; ANOVA was used to find out differences between cells in the same pH level and Student's *t*-test was used to find out differences within same cell between pH 7.2 and 6.5. (^∗^*P* < 0.05; ^∗∗^*P* < 0.01; ^∗∗∗^*P* < 0.001; ^§^*P* < 0.05; ^§§^*P* < 0.01; ^§§§^*P* < 0.001).

In order to further evaluate whether GPR4 having a regulation role in the expression of RANK/RANKL/OPG and the related molecular mechanism, gene knockdown and forced expression of GPR4 and the inhibitors of the downstream messenger molecules (cAMP and Ca^2+^) of GPR4 were used. The results showed that the gene and protein expression levels of RANK, RANKL, and OPG were significantly elevated in degenerated IVD-like acidic condition (Figure 3). SQ22536 (an adenylyl cyclase inhibitor preventing cAMP accumulation) and GPR4 knockdown abolished the upregulation in the expression levels of RANK, RANKL, and OPG in degenerated IVD-like acidic condition when compared to the control. GPR4 overexpression further promoted the upregulation of the gene and protein expression levels of RANK, RANKL, and OPG in degenerated IVD-like acidic condition when compared to the normal IVD-like acidic condition. U73122, an inhibitor of phospholipase C phospholipase C (PLC), could inhibit the release of Ca^2+^ from intracellular stores and plasma membrane Ca^2+^ inflow. The result of the current study demonstrated that U73122 had no significant influence on the expression levels of RANK, RANKL, and OPG in degenerated IVD-like acidic condition.

### 3.3. Involvement of GPR4 in the Upregulation of Neurotrophins in Degenerated IVD-Like Acidic Condition

**Figure 4:**
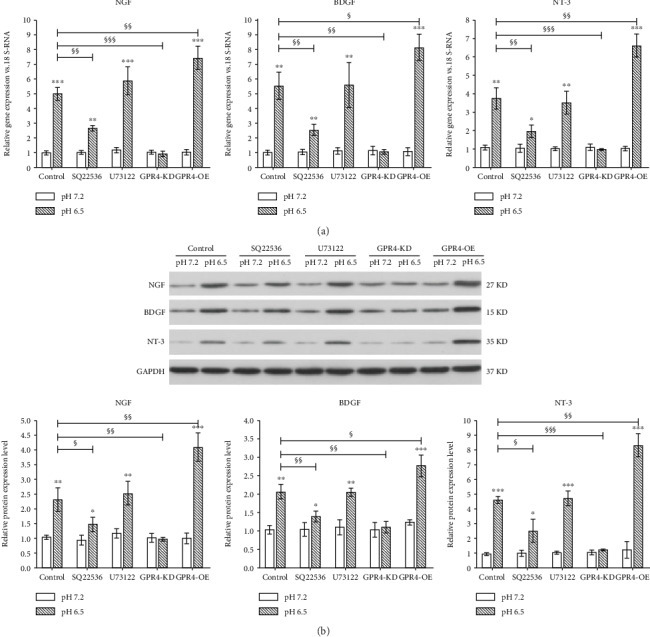
The influence of GPR4 and its downstream signaling molecules in the expression levels of nerve growth factor (NGF), brain-derived neurotrophic factor (BDNF), and neurotrophin-3 (NT-3) in the degenerated IVD-like acidic condition. The expression levels of NGF, BDNF, and NT-3 were assayed by RT-PCR (a) and western blot (b). Data were represented as mean ± SE of 6 independent experiments performed in triplicate; ANOVA was used to find out differences between cells in the same pH level and Student's t-test was used to find out differences within the same cell between pH 7.2 and 6.5. (NGF: nerve growth factor; BDNF: brain-derived neurotrophic factor; NT-3: neurotrophin-3; ^∗^*P* < 0.05; ^∗∗^*P* < 0.01; ^∗∗∗^*P* < 0.001; ^§^*P* < 0.05; ^§§^*P* < 0.01; ^§§§^*P* < 0.001).

To investigate the effect of degenerated IVD-like acidic condition on the expression of neurotrophins and the involvement of GPR4 in this process, the gene and protein expression levels of NGF, BDGF, and NT-3 were measured. As shown in Figure 4, the results of PCR and western blot showed the expression levels of NGF, BDGF, and NT-3 were upregulated in the degenerated IVD-like acidic condition when compared to the normal IVD-like acidic condition. The result also demonstrated that SQ22536 and GPR4 knockdown abolished the upregulation in the expression levels of all the three neurotrophins. On the opposite, GPR4 overexpression further promoted the upregulation in the expression of NGF, BDGF, and NT-3 in degenerated IVD-like acidic condition when compared to the normal IVD-like acidic condition. However, U73122 had no significant influence on the expression levels of neurotrophins in the degenerated IVD-like acidic condition.

### 3.4. Involvement of GPR4 in the Upregulation of Matrix-Degrading Enzymes in Degenerated IVD-Like Acidic Condition

**Figure 5:**
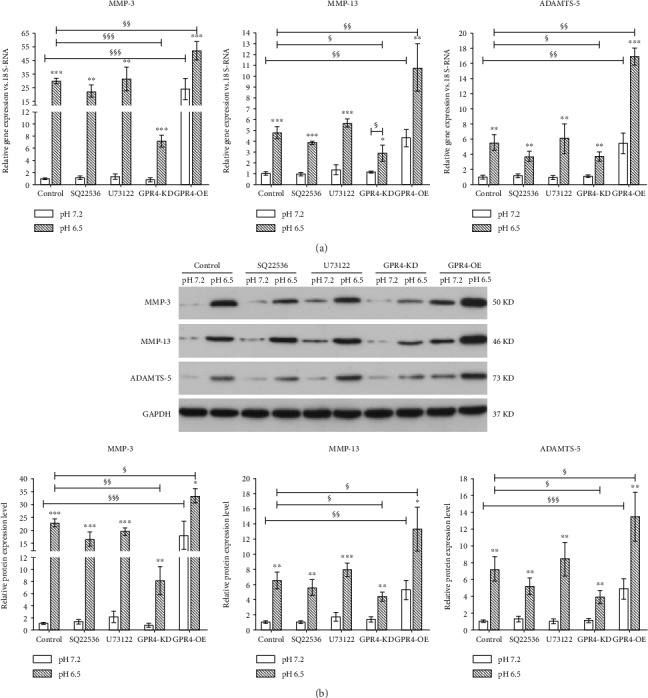
The influence of GPR4 and its downstream signaling molecules in the expression levels of matrix enzymes, including matrix metalloproteinase-3 (MMP-3), MMP-13, and aggrecanase-2 (ADAMTS-5) in the degenerated IVD-like acidic condition. The expression levels of MMP-3, MMP-13, and ADAMTS-5 were assayed by RT-PCR (a) and western blot (b). Data were represented as mean ± SE of 6 independent experiments performed in triplicate; ANOVA was used to find out differences between cells in the same pH level and Student's *t*-test was used to find out differences within the same cell between pH 7.2 and 6.5. (MMP-3: matrix metalloproteinase-3; ADAMTS-5: aggrecanase-2; ^∗^*P* < 0.05; ^∗∗^*P* < 0.01; ^∗∗∗^*P* < 0.001; ^§^*P* < 0.05; ^§§^*P* < 0.01; ^§§§^*P* < 0.001).

As RANK/RANKL/OPG could modulate the expression of matrix-degrading enzymes, including MMP-3, MMP-13, and ADAMTS-5, the expression levels of these enzymes were measured. As shown in Figure 5, we found the expression levels of MMP-3, MMP-13, and ADAMTS-5 in nucleus pulposus cells were all significantly increased in the degenerated IVD-like acidic condition, which were further promoted after GPR4 overexpression. Although, SQ22536 decreased the expression levels of MMP-3, MMP-13, and ADAMTS-5 in the degenerated IVD-like acidic condition, they did not abolish the upregulation. While, GPR4 knockdown abolished the upregulation in the expression levels of MMP-3, MMP-13, and ADAMTS-5 in the degenerated IVD-like acidic condition. Moreover, U73122 had no effect on the expression levels of the enzymes, which was similar to the effects of the inhibitors on the expression of RANK/RANKL/OPG.

## 4. Discussion

Microenvironment plays an important role in the maintenance of normal cell function and the development of diseases [28]. The acidic microenvironment is often observed in IVD, especially in the degenerated IVD. It is found that the pH range is 7.0 to 7.2 in the healthy disc and 6.5 to 6.8 in the mild degenerative IVD [17, 29]. Moreover, the acidic microenvironment is reported to be associated with the progression of IVD degeneration. For example, ASICs were reported to play critical roles in physiological and pathological conditions of the disc. Cai et al. found that the activation of ASIC1 resulted in cell apoptosis and stress-induced premature senescence of nucleus pulposus cells [18]. In another study, Gilbert et al. found that ASIC-3 promoted the catabolic and degenerate phenotype of nucleus pulposus cells [19].

In this study, we utilized degenerated IVD-like acidic conditions (pH 7.2 and 6.5 representing the acidic microenvironments of normal and degenerated IVD) to investigate the biological response of nucleus pulposus cells in the degenerated IVD-like acidic condition and find out whether GPR4 participated in this regulation effect. The result demonstrated that the presence of GPR4 was confirmed in nucleus pulposus cells and GPR4 was involved in the induced expression of RANK/RANKL/OPG system and neurotrophins under the degenerated IVD-like acidic microenvironment. This molecular mechanism may partly explain the reason why degenerated IVD-like acidic microenvironment could trigger the progression of IVD degeneration.

In recent years, a group of G-protein-coupled receptors, including GPR4, GPR65, GPR68, and G2A, have been identified as pH-sensing machineries that sense the change of the acidification in the microenvironments [30]. These pH sensors sense extracellular protons and stimulate different intercellular signaling pathways. According to previous studies, activation of GPR4 and TDAG8 mainly cause an increase in the second messenger molecular cAMP, while activation of GPR68 and G2A mainly elicit cytosolic Ca^2+^ [31, 32]. IVD is the largest avascular tissue in the body, which is characterized by nucleus pulposus cells residing within an acidic microenvironment. To the best of our knowledge, the expression of GPR4 in nucleus pulposus cells has not yet been detected in nucleus pulposus cells. In the present study, we found that GPR4 was expressed by the nucleus pulposus cells and the expression level was increased in the degenerated IVD-like acidic condition when compared to the normal IVD-like acidic condition. This indicated that GPR4 may participate in the triggering effect of degenerated IVD-like acidic condition on the progression of IVD degeneration.

Initially, RANKL was identified as a member of the TNF ligand superfamily and is well known to regulate bone metabolism [33]. RANKL interacts with RANK, which is expressed on the membranes of mature osteoclasts and osteoclast precursors, to promote differentiation and activation of osteoclasts. It is reported that the RANK/RANKL/OPG system may play a part in the process of IVD degeneration; RANKL can induce the expression of genes encoding inflammation mediators [10, 11]. In the study of Okito et al., they found that GPR4-mediated RANKL expression of osteoblasts in an acidic microenvironment via the cAMP/PKA signaling pathway [25]. We therefore focused on the relationship between GPR4 and the expression of RANK/RANKL/OPG system in the degenerated IVD-like acidic condition. Compliance with previous findings, the results of the current study showed that GPR4 was involved in the increased expression of RANK/RANKL/OPG in the degenerated IVD-like acidic condition. In addition, among the three components of the RANK/RANKL/OPG system, the expression of RANKL was most strongly stimulated by acidic stimulation and GPR4 overexpression. These data suggested that GPR4-mediated RANKL upregulation in nucleus pulposus cells in the degenerated IVD-like acidic condition, which activated the inflammation in degenerated IVD.

Previous studies have suggested that the expression of MMP-3 and MMP-13 by nucleus pulposus cells was significantly upregulated by exogenous RANKL in the presence of IL-1*β* [11]. This result led us to analyze whether GPR4 have the potential to regulate the expression of MMP-3, MMP13, and ADAMTS-5 in the degenerated IVD-like acidic condition, as these three enzymes are the main catabolic factors of the extracellular matrix of IVD. We found that the expression of MMP-3, MMP13, and ADAMTS-5 was elevated in the degenerated IVD-like acidic condition, which can be promoted or attenuated by GPR4 overexpression or knockdown, respectively. This finding was similar to the expression of RANK/RANKL/OPG system. Based on this result, we speculate that degenerated IVD-like acidic condition may promote RANKL expression via GPR4, and then induce the expression of these catabolic enzymes. However, after GPR4 knockdown and inhibiting cAMP accumulation, these catabolic enzymes could still respond to acidity stimuli, suggesting other activation pathway existence. It indicates where further investigation is required.

Many studies have described that sensory fibers are present in the outer layers of the annulus fibrosus under normal conditions [34, 35]. And in animal models, the induction of nerve ingrowth was reported to be associated with the progression of IVD degeneration [35–37]. It is found that disc cells themselves produce a number of different neurotrophins that are active in neurite survival and outgrowth. These neurotrophins include NGF, BDGF, and NT-3, which are overexpressed in response to IL-1*β* and TNF*α* [37]. The present study demonstrated that the expression levels of NGF, BDGF, and NT-3 were increased in the degenerated IVD-like acidic condition when compared to the normal IVD-like acidic condition. A more important and highly relevant concept is that GPR4 was involved in the upregulation of neurotrophins in degenerated IVD-like acidic condition. This result indicated that at least in part, GPR4 mediated the elevated expression of NGF, BDGF, and NT-3 in the degenerated IVD-like acidic condition.

Previous study shows that GPR4 activation by acidosis could stimulate cAMP/PKA signaling and PLC/Ca^2+^ signaling. To further identify the downstream signaling that mediates the action of GPR4, we subjected nucleus pulposus cells with the inhibitors of SQ22536 and U73122, which inhibited the downstream cAMP accumulation and Ca^2+^ mobilization, respectively. Our study revealed that only SQ22536 could abolish the upregulation in the expression of both the RANK/RANKL/OPG system and neurotrophins (NGF, BDGF, and NT-3). This finding was consistent with a previous study that said the activation of GPR4 mainly causes an increase in the intracellular cAMP [38, 39]. Taken together, these results indicated that GPR4 participated in the regulation of the expression of RANK/RANKL/OPG system and neurotrophins via cAMP accumulation, but not Ca^2+^ mobilization.

There were limitations in this study. Firstly, the in vitro experiment could not totally mimic the microenvironment and cellular interaction *in vivo* of IVD. Second, we did not investigate all the molecules and signaling which may be related to the effect of GPR4. However, the findings in this study could provide a new therapeutic strategy to mitigate the development of IVD degeneration by targeting the GPR4 sensation and downstream pathway.

## 5. Conclusion

In this paper, we found that, at least in part, GPR4 was involved in the increased expression of RANK/RANKL/OPG system and neurotrophins by nucleus pulposus cells under the degenerated IVD-like acidic microenvironment.

## Figures and Tables

**Table 1:** Primers used for RT-PCR.

Gene	Primer sequences
GPR4	F: 5′-CGGGTCAAGACAGCAGTAG-3′
	R: 5′-ACAAGGTGTGGTTAGCGAT-3′

RANK	F: 5′-ACGTGGACCCTTGCCCCAGT-3′
	R: 5′-ACTGGCCACCAGGGGAGCTT-3′

RANKL	F: 5′-GACAGGCACGGACTCGTA-3′
	R: 5′-CGCTCATGCTAGTCGTCTA-3′

OPG	F: 5′-TACAGCATCACTACGTAGGAC-3′
	R: 5′-ACGTCATGCGATCACATATCG-3′

NGF	F: 5′-GCCCACTGGACTAAACTTCAGC-3′
	R: 5′-CCGTGGCTGTGGTCTTATCTC-3′

BDGF	F: 5′-GGTCACAGTCCTGGAGAAAG-3′
	R: 5′-GTCTATCCTTATGAACCGCC-3′

NT-3	F: 5′-GATCCAGGCGGATATCTTGA-3′
	R: 5′-AGCGTCTCTGTTGCCGTAGT3′

MMP-3	F: 5′-CCTCTGATGGCCCAGAATTGA-3′
	R: 5′-GAAATTGGCCACTCCCTGGGT-3′

MMP-13	F: 5′-TGACTATGCGTGGCTGGAA-3′
	R: 5′-AAGCTGAAATCTTGCCTTGGA-3′

ADAMTS-5	F: 5′-AGTTTGCCTACCGCCATTG-3′
	R: 5′-CATTCTGTCCCATCTGTAACCTT-3′

18S-RNA	F: 5′-GAATTCCCAGTAAGTGCGGGTCATA-3′
	R: 5′-CGAGGGCCTCACTAAACCATC-3′

**Table 2:** DNA sequences GPR4-shRNA and scramble sequence.

Target	Sequences (5′-3′)
shRNA1	AGGAGAAAGTCAAGATCAAAC
shRNA2	TCTTTCATGACGAGCTCTTTC
shRNA3	TAGCCTTCACCAGCCTCAATT
Scramble sequence	CCTAAGGTTAAGTCGCCCTCG

## Data Availability

The data used to support the findings of this study are included within the article.
